# The Rational Treatment of So-Called Inoperable Uterine Cancer

**Published:** 1910-05

**Authors:** Walter B. Chase

**Affiliations:** Brooklyn, New York. Visiting Surgeon Bethany Deaconess’s Hospital; Consulting Obstetrician and Gynecologist to the Long Island College Hospital; Consulting Gynecologist to the Nassau Hospital, and the Jamaica Hospital


### The Rational Treatment of So-called Inoperable Uterine Cancer

By WALTER B. CHASE, M. D., Brooklyn, New York.
Visiting Surgeon Bethany Deaconess's Hospital; Consulting Obstetrician and Gynecologist to the Long Island College Hospital; Consulting Gynecologist to the Nassau Hospital, and the Jamaica Hospital.

THE treatment of uterine cancer when it has reached the so-called inoperable stage, seems in the vast majority of cases, to be limited to the vaginal douche and the internal administration of opiates. These two expedients appear to most practitioners as affording all the relief they are able to bestow on its unfortunate possessor. When it is remembered that reliable statistics of registration areas show that one woman in eleven dies of cancer, and that after the age of thirty-five the mortality reaches one in nine, the magnitude of its destructive influence and the distress and suffering it occasions stands forth in all its hideous proportions, not taking into account the deaths from this cause which are not correctly reported. It is safe to affirm that no other disease makes its unfortunate possessor so unbearable to herself and her friends, and inflicts such terrible suffering on its unfortunate victim. It seems incomprehensible, when the sympathy and resources of the public are laid under contribution to most other diseases, that these cases go on to irremediable death without systematic humane effort to administer to their necessity, either in hospitals or homes, save when the possession of ample resources makes such care possible: What can be done for these unfortunates? First, the humane sentiment of every community, if wisely guided, should provide hospitals and care for these neglected cases, as certainly will be done when enlightened public opinion is directed in proper channels. What then in the light of present knowledge may be done, medically or surgically, for these cases?

First, the use of the thermo-cautery; second, the use of radium; third, the application of the Röntgen ray; and fourth, the daily local aseptic dressing.

The writer believes that the suffering of cancer is due principally to the pressure of the malignant growth on incarcerated nerve structures, and to the want of cleanliness of ulcerating surfaces; and that relief comes from the removal of all diseased tissue as far as possible, and persistent cleansing of ulcerating surfaces.

The objection to strong escharotics in the treatment of cervical or corporeal cancer, is first the impossibility of limiting its corrosive action, and second, the atrocious and persistent suffering occasioned. It is in this connection that the use of a thermocautery is of the highest value.

*First.*—If done as it should be under anesthesia, there is commonly little or no pain following its use.

*Second.*—Through heat on the parts invaded by the disease, short of actual disintegration there is evident inhibition if not destruction of pathogenic germs.

*Third.*—The absorbent vessels are effectively closed.

*Fourth.*—The systemic infection is usually diminished—sometimes disappearing for a longer or shorter period of time.

*Fifth.*—In occasional cases (more often of cervical cancer) unexpected recovery has followed its use.

The statistics of the late Dr. John Byrne, of Brooklyn, show that in 367 cases of uterine cancer (not selected cases) operated on by the thermocautery, at the end of five years over 19 per cent. of cases were yet alive; unequaled it is believed by the statistics of any other operator by any other method, even on selected cases. The writer's results are equally satisfactory. An added value of this form of treatment lies largely in the fact it can be reapplied from time to time as is deemed expedient. Cases of this class followed by daily dressing with oxydzinc gauze, and gentle irrigation with permanganate of potassium, or the creasol compounds, may be carried to the end with greatly diminished, and in some instances, with but little pain. Besides the exhausting influence of repeated hemorrhage is much diminished, and the general comfort of the patient promoted.

In hospitals the electro-cautery can usually be installed and used with proper platinum knives with great satisfaction; though the Pacquelin-cautery with similar platinum accessories can be used with effectiveness, and outside of hospital practice one is usually restricted to the Pacquelin-cautery. Tact and skill in the use of a proper speculum so as not to impinge on diseased surfaces (often the Sims with the patient in the Sims position) and the parts protected from burning by asbestos paper will make possible the safe removal of diseased structures without troublesome hemorrhage, and include the destruction of tissues not removable with a knife or by the use of caustic.

Boldt has devised a tubular speculum for the use of the thermo-cautery, so constructed that a stream of cold water traverses the hollow spaces in the speculum preventing risk of burning of the vaginal wall.

In a general way it may be stated, that nearly every case of cervical or uterine cancer, save in the final stage, may be treated by the thermo-cautery, with advantage and resulting comfort. The more diseased structure removed the greater the benefit. The first results are diminution of pain, lessened discharge, and a healthier appearance of granulating surfaces. As a rule, if there is no burning of the muco-cutaneous surfaces, there is little or no pain as a result of the operation. The critical question is the extent to which the diseased tissue can be removed without injury to the bladder, ureters, and the intestinal tract. It has not been my misfortune to injure any of these structures by the use of the cautery, though the possibility of it should be kept in mind, and explained to the friends in advance of the operation, for such an accident might be no evidence of lack of skill on the part of the operator. The accident to my knowledge has happened to one of the most distinguished operators of our country. Dr. Byrne states he had no *primary mortality from the operation.*

I believe, with due care, the risk is so little as to be practically negligible. Some prerequisites are necessary, perfect anesthesia, capable assistants, proper apparatus, adequate protection of the vagina from burning, great patience in the progressive steps in the application of the knife at a dull red heat, so as not to burn the tissues so rapidly as to provoke hemorrhage, and to cook it sufficiently to destroy as far as possible malignant structures, and the exercise of judgment and skill in the area of structures to be removed. With care and experience it is frequently possible to remove all of the cervix, and most of the body of the uterus (when involved) save a little more than a shell of the peritoneum.

The nature of the attack will be determined by the extent of the disease. If the cervix is intact (or nearly so), it may be seized by a strong double volsella for purposes of downward traction, or if insufficient for this purpose a strong double tanaculum, opening outwards can be introduced within the cervical canal for a similar purpose. In applying the cautery knife, the incision encircling the cervix should, if possible, be above the growth, and if need be reaching to the utero-vaginal junction. The traction should be even and constant while the cautery knife slowly eats its way through the tissue near the circumference of the uterine body, but inside the peritoneal covering. To make the cautery knife effective, it should be kept in slow motion up and down or laterally, otherwise it is likely to provoke hemorrhage and delay progress, at the same time keeping it free for the eschar. If hemorrhage happens, which is always a troublesome possibility, a small gauze sponge saturated with diluted acetic acid, or of a solution of adrenalin carefully applied with pressure, usually controls the bleeding until the slow reapplication of the cautery closes the bloodvessels. If after all that has been accomplished by this step has been done, and it is found that infected portions of intrauterine structure remains, a round, dome-shaped instrument can be cautiously introduced into the uterine canal and further cauterising be facilitated. So also can infected areas of disease on the vaginal wall be removed by the curved platinum knife. When the disease is far advanced and much vaginal involvement is present, the cancerous structure should as far as possible be removed by the same method, but by slow progressive steps, or the application of a strong curet may be applied following with the cautery knife. Even in these later stages enough of the involved structure can be removed to quiet or obtund pain, check or control hemorrhage, and lessen in greater or less measure the discharge from diseased surfaces, thereby diminishing the suffering and the rapid tendency to exhaustion. Daily irrigation and dressing of the parts must be carefully persisted in.

As yet the exact status of the value of radium is not known. While my personal experience is insufficient to enable me to speak *ex cathedral,* of its safety and value in certain cases I am thoroughly convinced. Its power to diminish the proliferating cell growth is undoubted, and that malignant structures under its use with radium of a relatively high radio-activity gives hope that much that has been claimed for it will prove true. Unfortunately, its high cost almost precludes its use.

Regarding the efficacy of the *x*-ray treatment in carcinomatous disease there seems to be a wide diversity of opinion. In my personal use of this agent in uterine cancer, I am not wholly decided, though in cases for a time benefits seem to follow its use. While it is true that proper application of the agents mentioned should not be carried beyond conservative limits, the fact remains that their wise use guided by discrimination and judgment are at present, the best agents we have to combat this disease, and who will dare to affirm that the end to be gained not only justifies the means, or that for humanity's sake this method of treatment should not become general?

Following or coincident with the use of these several remedies the mixed serum of erysipelas and prodigrosus may be employed. Coley says it acts to inhibit the recurrence of carcinoma.

While study and investigation give hope that serum therapy will bring a radical cure, there is *ad interim,* every reason why the diffusion of knowledge among women should be inculcated by every legitimate method, until physicians and women are alike awake to the strategic importance of early investigation and accurate diagnosis of conditions which demand careful scrutiny and perhaps the assistance of some one especially fitted by observation, study and experience in the advancement of these cases, in the hope that early radical measures may anticipate the inevitable approach of the period when all but palliative treatment is impossible. So long as procrastination supplants the imperative need of prompt and exact diagnosis, so long will the roll of mortality tell its tale of hopeless woe. Added to this the time is ripe, when in every community, some plan of concerted action should be adopted in which the medical profession, public-spirited citizens, and intelligent women should in some appropriate and effective way, commence a system of education among women whereby they should be informed of those symptoms which make absolutely imperative such examination as would reveal or exclude the early symptoms of malignancy. If the lethargy of indifference on the part of some of the medical profession, and the false security of women which such symptoms as have been enumerated, could make way for concerted action on the part of both, to know the truth and that early, a new hope would spring in hearts which are now utterly sad. This is the crux of the whole subject. Every investigation, intelligently and thoroughly pursued, to the end that radical treatment might be undertaken in suitable cases or when such a period has passed the use of palliative treatment as will afford the greatest possible relief from suffering. If space permitted I would give more in detail the results of my own experience in treating these cases, but will refer to three cases, which I copy from a paper I read on this subject, before the Section of Obstetrics and Gynecology of the American Medical Association, at Atlantic City, June, 1909, and published on December 4, 1909, in the *Journal of the American Medical Association.* In the discussion following the paper participated in by Kelly, Boldt, Frederick, Stone, Polak, Wetherill, andothers, the fact becomes apparent that the use of the thermo-cautery as recommended and so successfully practised by Dr. Byrne is becoming recognised as the most effective method of treating uterine cancer when the period for radical measures has passed. Further there was evidence of a sentiment favoring the diffusion of specific knowledge among women of the early symptoms attending the presence of uterine cancer. The first two cases herewith reported were of the same type of carcinoma and instructive in showing the modifying influence of palliative treatment by the thermo-cautery. In none of the cases was any hope held out to the patient of ultimate cure.

Case I.—The patient, a married woman, aged 43, entered the Skene Sanitarium, in March, 1901, with a cauliflower excrescence of the cervix as large as a man's fist. She had had numerous conceptions all of which terminated in miscarriages; but as these were intentionally procured there was a well grounded suspicion that her disease was related to the incident traumatism. This growth was reflected on the vaginal wall antero-posteriorly and laterally, which forbade a primary effort at hysterectomy. On March 14, I removed the growth by the galvano-cautery and amputated the cervix at the vaginal junction. The surfaces healed kindly, save a small area on the uterine stump about the size of a silver half-dollar. On May 21, following, the patient re-entered the sanitarium and I performed an abdominal hysterectomy. Prior to this operation she was weak, anemic and in poor physical condition. Her convalescence was satisfactory. She was kept under monthly observation by her physician, and in May, 1902, a year later, there appeared at the seat of the vaginal cicatrix a nodular mass rather larger than a silver twenty-five cent piece. At this time she entered the Memorial Hospital and I removed a button of tissue opening from the vagina into the peritoneal cavity, the size of a silver half-dollar. I examined her during the past year and she is in perfect health, with no sign of the return of the growth.

Case II.—The patient, a married woman, aged 46, German, multipara, dated her trouble to a miscarriage, seventeen years previously. She had a large, bleeding, ulcerating cauliflower excrescence of the cervix, extending to the vaginal walls, which nearly filled the vagina. The discharge was offensive and the patient's health was seriously impaired from the frequent hemorrhages, associated with marked cachexia. She entered the Memorial Hospital, September 23, 1902, and on the 25th I removed the entire growth by the thermo-cautery. In two months' time it had healed under daily dressings, save for a cup-shaped cavity three-quarters of an inch in diameter and one-half inch deep. The patient's general health was greatly improved. Owing to non-healing she had two more thermo-cautery operations in November, 1902, and June, 1903. In November, 1903, the disease having made some progress without materially affecting the patient's general health, Röntgen ray apparatus was installed in the home and on every second or third day for nearly three months treatment was given. This was followed by the use of radium and the ulcerative process was greatly retarded, and the patient's general health fairly maintained with but slight pain, up to the time of her death, May 7, 1904. For several weeks previous to her death the normal anatomic relations between the vagina, bladder and rectum were altogether lost and the amount of gauze daily used to fill this cavity amounted to many yards. During the twenty-one months of attendance the patient was visited by myself or my assistants about 750 times. It is at once apparent that such attention is most exacting in its demands on the attendant, but it demonstrated the fact that almost entire freedom from pain was the result, and that it was worth all it cost.

Case III.—During March, 1896, a married woman, multipara, aged 42, came under my observation with typical cancer of the cervix, accompanied with extensive involvement. Hemorrhage was violent and the patient was cachectic. She was greatly exsanguinated and very weak. She entered St. John's Hospital in March, and I did a high galvano-cautery amputation as soon as her health permitted. She made a slow but satisfactory recovery, as far as the healing and local symptoms were concerned, and after two or three months she was able to resume her family duties. In November of the same year she entered the Bushwick Hospital for extirpation of a large gland of Bartholin. At this time there was no sign of return of the cancerous growth. On June 16, 1897, she re-entered the Bushwick Hospital, being seven months pregnant. The disease had returned, springing up around the old stump. After watching its behavior, I feared, from the hardening and infiltration of the uterine and contiguous structures, labor might induce rupture of the uterus, and on July 18, at the eight month of pregnancy, I removed the diseased growth, which encircled the uterine outlet, by the thermo-cautery. No shock followed and the patient was delivered of a healthy living child on August 6. Her convalescence from the confinement was satisfactory as was the healing after the cautery. She enjoyed good health for nearly a year. Then the growth reappeared and she entered the Central Hospital, June 21, 1898, and I removed as far as possible the cancerous mass which had returned. She returned home August 25. The healing was not satisfactory and she died a few weeks later from cerebral embolism, which only anticipated the inevitable results of her condition.

It is my hope in the not far distant future to lay before the medical profession a more comprehensive discussion on the subject of so-called inoperative uterine cancer, in the hope it will facilitate such a dissemination of knowledge as will lead to an earlier and more effective management of these most unfortunate cases.

Apropos to this I append a brief abstract of my paper read before the Medical Society of the State of New York, January 26, 1910, entitled "The Duty the Medical Profession Owes the Woman with Cancer."

Whereas the appalling fact that statistics of registration areas show that one out of eleven of all women die of cancer, and that after reaching the age of thirty-five years, the mortality increases to one in nine; and that as most of these are cases of uterine cancer, the question arises, whether the medical profession does not owe an unfulfilled obligation to this most unfortunate class of sufferers.

While large benefactions and well organised efforts are ministering to the comfort of those suffering from tuberculosis, there is in this State no systematic humane plan to reach those more terrible cases, condemned to helpless suffering and torturing death.

There is substantial basis for the belief, that earlier recognition of the presence of malignancy, in these cases, would add something to the measure of relief to be obtained by radical or palliative treatment. Among these unfortunate women, are large numbers, whose resources make it impossible for them to secure proper medical advice or capable nursing. It is also painfully apparent that home and hospital facilities for incurables is entirely inadequate to meet the needs of society. It is farther a well demonstrated fact that appropriate palliative treatment, carefully and systematically persisted in, will relieve in larger measure the suffering of those doomed to certain death.

In view of these facts it is resolved, first, that the Medical Society of the State of New York, by its President, shall appoint a committee of five whose duty shall be to urge on all practitioners of medicine, in this State, greater care in making early diagnosis in cases of suspected uterine cancers. Second, resolved, that this committee be directed to devise some method, by which along ethical lines, women may be properly informed as to the reason why they should seek early professional advice in menstrual and hemorrhage disorders, and that they are farther instructed to consider some more comprehensive plan, whereby a general diffusion of appropriate and vital knowledge may be promulgated on this very important subject. Third, resolved, that this committee be directed to report its recommendations at the next meeting of the society. Fourth, resolved, that the treasurer of this society be directed to honor payment of bills incurred for printing and needful correspondence if not otherwise provided for), and that this committee be empowered to fill vacancies in its membership and appoint sub-committees if deemed expedient.

1050 Park Place.

---

"So you think worry kills more people than work?"
"I'm sure of it," replied the sarcastic scientist.
"Why?"
"Because so many people find it easier than work and devote their time to it."—*Washington Star.*

